# Movement Coordination or Movement Interference: Visual Tracking and Spontaneous Coordination Modulate Rhythmic Movement Interference

**DOI:** 10.1371/journal.pone.0044761

**Published:** 2012-09-17

**Authors:** Veronica Romero, Charles Coey, R. C. Schmidt, Michael J. Richardson

**Affiliations:** 1 Center for Cognition, Action and Perception, Department of Psychology, University of Cincinnati, Cincinnati, Ohio, United States of America; 2 Department of Psychology, Colby College, Waterville, Maine, United States of America; 3 Department of Psychology, College of the Holy Cross, Worchester, Massachusetts, United States of America; McMaster University, Canada

## Abstract

When an actor performs a rhythmic limb movement while observing a spatially incongruent movement he or she exhibits increased movement orthogonal to the instructed motion. Known as rhythmic movement interference, this phenomenon has been interpreted as a motor contagion effect, whereby observing the incongruent movement interferes with the intended movement and results in a motor production error. Here we test the hypothesis that rhythmic movement interference is an emergent property of rhythmic coordination. Participants performed rhythmic limb movements at a self-selected tempo while observing a computer stimulus moving in a congruent or incongruent manner. The degree to which participants visually tracked the stimulus was manipulated to influence whether participants became spontaneously entrained to the stimulus or not. Consistent with the rhythmic coordination hypothesis, participants only exhibited the rhythmic movement interference effect when they became spontaneously entrained to the incongruent stimulus.

## Introduction

A central question in the study of joint action and social coordination concerns how the observed movements of other individuals and objects in the environment influence the production of one's own actions. If an individual observes a conspecific smiling, touching their face or shaking their foot, for instance, then that individual also tends to, smile, touch their face or shake their foot [1], [2]. Several researchers have theorized that such phenomena are the result of the pre-motor cortex representing action execution and observation simultaneously and in a common code [3]–[6]. The discovery of ‘mirror neurons’ – cells in the pre-motor cortex of Macaque monkeys that fire during both execution and observation of a given action – coupled with subsequent research that suggests an analogous system in the human brain [see 7 for a review], is argued to provide strong support for this neural-cognitive account. That is, observing the behaviors of someone else is thought to activate the neural structures involved in the production of that same action. Further empirical support for this ‘motor resonance’ hypothesis has come from behavioral studies demonstrating that observing the actions or movements of other individuals or environmental stimuli can result in movement imitation and mimicry, as well as movement modification, perturbation and onset delay, [8,9].

One such behavioral phenomenon argued to provide support for the above hypotheses is *rhythmic movement interference* (RMI). RMI refers to the increased movement fluctuations that occur in the orthogonal (i.e., non-instructed) movement direction when an individual is instructed to produce rhythmic movements in a horizontal or vertical direction while observing a spatially incongruent movement (e.g., watching a horizontal movement while producing a vertical one). Several studies in which participants are instructed to perform either horizontal or vertical rhythmic arm movements while observing a stimulus moving in either the congruent or incongruent dimension have demonstrated this effect [10]–[13]. RMI is assessed by inspecting the variance of the participant's movements in the orthogonal or non-instructed direction of motion (e.g., variability in the vertical plane while producing horizontal rhythmic movements). It is considered to have occurred when this variance is greater for conditions in which the participants observed incongruent compared to congruent movements. Standard accounts of this phenomenon interpret RMI as ‘motor contagion’ [e.g.], [14,12], wherein observing an incongruent stimulus movement activates the neural structures that correspond to the production of movement in the same spatial dimension as the stimulus. The observed movement thus *interferes* with production of the intended on-going movement (i.e., RMI).

There is some evidence, however, that suggests that rhythmic movement interference should not be framed in terms of “interference” or “motor error”, but rather as the result of the dynamical entrainment processes known to constrain rhythmic movement coordination [15]. Specifically, Richardson et al. (2009) conducted a standard RMI experiment in which participants were instructed to coordinate their arm movements with either congruent or incongruent movements produced by a confederate. Through an examination of the time-evolving structure of the variability in the participants' non-instructed movements, Richardson et al. (2009) demonstrated that RMI is not ‘noise’ or the result of error-like fluctuations. Rather, the non-instructed movements contained coherent oscillations that were actually entrained to the observed movements of the confederate. Moreover, the Richardson et al. (2009) findings were consistent with past research demonstrating that the observed patterns of rhythmic movement coordination are constrained by the self-organizing dynamics of coupled oscillators [see 16].

Motivated by the dynamical systems approach to human behavior [17]–[19], the Richardson et al., (2009) *rhythmic coordination* hypothesis suggests that RMI should be understood as an emergent and constructive property of coupled perception-action systems. That is, the rhythmic coordination hypothesis suggests that RMI may be a result of the dynamic processes that constrain both intra- and interpersonal limb movements to behave in a synergistic and coordinated fashion [20]–[26]. Two previous findings from research on rhythmic coordination are of particular significance. First, ample research has demonstrated that the limb movements of two co-actors, or the limb movements of an individual and an environmental rhythm (e.g., a computer-generated oscillatory stimulus), will become spontaneously coordinated provided there is an informational visual coupling strong enough to overcome any inherent differences between the movements involved—for instance, different natural movement frequencies or other spatial-temporal characteristics [27]–[29]. Second, research has demonstrated that the recruitment of additional movement degrees-of-freedom is an essential source of task-specific flexibility, whereby previously quiescent degrees-of-freedom are spontaneously employed in response to changes in task demands or in order to stabilize movement coordination [30,31].

As a specific example of this latter point, Fink, Kelso, Jirsa and De Guzman (2000) demonstrated that the recruitment of additional degrees-of-freedom increases “coordinative flexibility” during rhythmic interlimb coordination, allowing participants to sustain a pattern of anti-phase coordination under frequency conditions in which that pattern would typically have become unstable had those degrees-of-freedom not been employed. Participants were required to produce rhythmic wrist movements in an anti-phase coordination pattern and steadily increase the frequency of oscillation. Anti-phase coordination refers to the pattern of rhythmic coordination that occurs when two movements oscillate back and forth at the same time but in opposite directions and is known to be less stable than in-phase coordination (i.e., movements oscillate back and forth at the same time and in the same direction), becoming unstable at fast movement frequencies [32]. With respect to the latter point, numerous studies have demonstrated how participants spontaneously transition from anti-phase to in-phase coordination as movement frequency is scaled beyond a critically fast frequency [17]–[20]. Fink et al. (2000) demonstrated, however, that this transition only reliably takes place when participants are constrained to produce movements in the primary movement direction alone (i.e., are constrained to single degree-of-freedom movements). When participants were unconstrained and were also free to move in an orthogonal direction (i.e., employ other degrees-of-freedom) they very rarely switched from the anti-phase to the in-phase pattern. An examination of the movement trajectories revealed that in the unconstrained condition participants' movements significantly deviated from the instructed axis of motion and that this deviation was greater at faster movement frequencies. This spatial deviation revealed how the recruitment of extra degrees-of-freedom provided participants with the flexibility to stay in an anti-phase mode of coordination for longer than would have been sustainable when constrained to only one degree-of-freedom.

Collectively, the above coordination findings anticipate the rhythmic coordination hypothesis proposed by Richardson et al. (2009) and provide corroborating evidence that increased non-instructed movement during incongruent movement coordination is a constructive process and not an ‘error’ in movement production. Indeed, these findings support the proposal that RMI is evidence of how the multiple degrees-of-freedom of a limb or body movement are organized by the neural, biomechanical, informational and environmental constraints of a given task structure to behave as a functional synergy or coordinative structure [33,34].

A central prediction of the rhythmic coordination hypothesis is that the RMI effect should only be observed when the instructed movement becomes entrained to the observed movement. If RMI is in fact the result of the movement system spontaneously recruiting additional degrees-of-freedom to sustain coordination with an incongruent observed movement, then RMI should only occur during coordination with the observed movement, as opposed to simply attending to the incongruent movement in absence of coordination. The current study was designed to test this prediction by investigating whether the RMI effect is dependent on the degree to which individuals become spontaneously coordinated with the observed movement. All of the previous research on RMI has instructed participants to coordinate with the observed movement or stimulus. The specific question addressed here is whether RMI occurs when individuals observe an incongruent movement and spontaneously coordinate with it, even though they are not instructed to do so.

Essential to the testing of this rhythmic coordination prediction was (1) to ensure that participants did not intentionally coordinate with the observed stimulus (i.e., coordination should be unintended) and (2) to manipulate the strength of visual coupling, while ensuring that participants are always able to observe the movements of the congruent or incongruent stimulus. Both of these conditions were fulfilled by adapting the spontaneous environmental coordination paradigm developed by Schmidt et al. (2007), and employed in several subsequent studies [35,27], to demonstrate how an individual's rhythmic limb movements can become spontaneously entrained to the movement of a visual stimulus and that the degree of entrainment observed is mediated by the strength of the informational coupling (i.e., visibility/attention). In this paradigm, participants were instructed to read letters that appeared at random intervals either on or above an oscillating visual stimulus, while simultaneously performing a rhythmic limb movement. The spontaneity of coordination was achieved by informing participants that the experiment was investigating the effects of irrelevant motor activity on symbolic processing, and by instructing participants to produce limb movements at a self-selected tempo. The strength of the visual coupling was manipulated by having the letters displayed on the oscillating visual stimulus, whereby participants needed to visually track the stimulus in order to read the letters (which results in stronger entrainment), or just above the center of the stimulus's motion, whereby participants observed the entirety of stimulus movement but had no need to track it (which results in weaker entrainment). Finally, to ensure that the coordination with the stimulus was spontaneous the participants were asked questions about what they thought the experiment concerned and if they noticed whether they were coordinated with the stimulus before being fully debriefed.

## Materials and Methods

### Participants

Twelve undergraduate students from Colby College (N = 8) and the University of Cincinnati (N = 4) participated in the study for partial course credit.

### Task and materials

**Figure 1:**
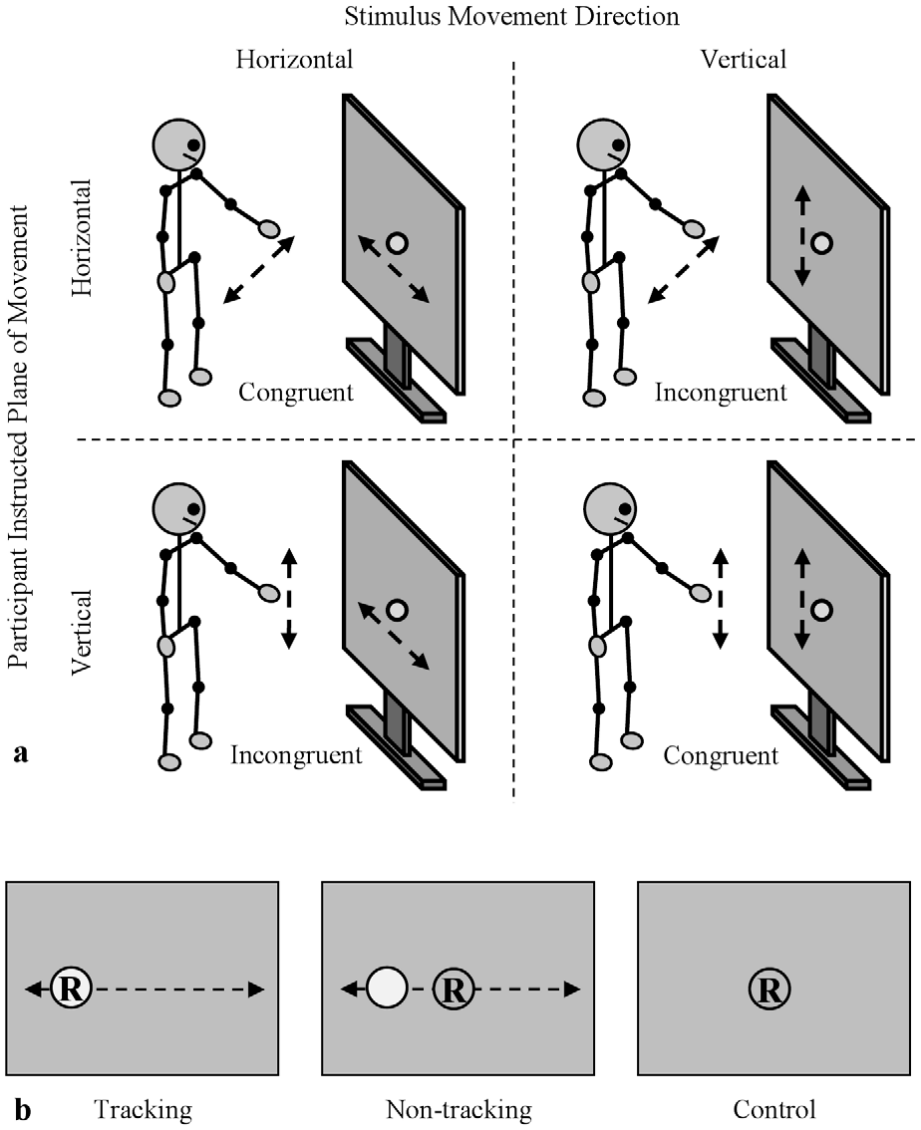
Experimental setup. (a) different congruency conditions (instructed participant direction×stimulus direction). (b) Illustration of the two visual tracking conditions and the control condition (horizontal stimulus motion depicted).

Participants performed movements of the right-forearm (right index finger extended) in the horizontal (frontal) and vertical (sagittal) dimensions, at a self-selected comfort-mode tempo, while observing a visual stimulus that oscillated horizontally or vertically on a rear projection screen. Participants stood facing the projection screen with the tip of their index finger 75 cm away from the center of the screen (Figure 1a). The oscillating visual stimulus was a small red dot (diameter of 5 cm) that oscillated both vertically and horizontally with a frequency and amplitude of 0.8 Hz and of 40 cm, respectively. The stimulus was projected so that the center of its motion was aligned with the central position of the participant's extended forearm.

Visual tracking was manipulated by instructing participants to read aloud letters that were presented at random intervals either on the oscillating visual stimulus (tracking condition) or on a stationary visual stimulus located at the center of the oscillating stimulus's motion (Figure 1b). Participants also performed a set of control trials, in which no oscillating visual stimulus was presented and participants simply oscillated their forearm horizontally or vertically while reading aloud letters presented on the centrally positioned stationary visual stimulus. Although no oscillating stimulus was presented for these control trials, a stimulus motion time-series was still generated so that chance level coordination could be determined. For all conditions the letters were presented for 250 ms with random inter-stimulus intervals between 1500 and 3000 ms.

A small 1×.5 cm sensor was fixed to the tip of the participant's right index finger and their movements were recorded at 60 Hz (0.01 mm spatial resolution) using a Polhemus magnetic motion tracking system (Polhemus Ltd, VT). The Polhemus emitter was positioned just behind the participant's right arm at waist height with the X-axis pointed directly toward the projection screen. This positioning ensured that predominantly horizontal limb movements were captured as variability in the position of the sensor in the Y-axis, while predominantly vertical limb movements were captured as variability along the Z-axis. In turn, non-instructed movement was assessed with respect to the variability in the opposite dimension; variability in the Z-axis during horizontal instructed movements and variability in the Y-axis during vertical instructed movements. The alignment of the emitter with respect to participants' limb position and movement direction was checked between trials to minimize the possibility that deviations in the orthogonal direction of movement were due to misalignment. A single PC computer was used to simultaneously present the stimuli and record participant movements.

### Procedure

Upon arrival, participants were informed that the experiment was investigating the effects of movement on symbolic processing and that they would be required to produce and observe distracter movements while reading aloud letters that were flashed at random intervals on a projection screen. As mentioned above, this cover story was employed to ensure that participants were naive to the study's true purpose and that any coordination that resulted was spontaneous^1^. The experimenter demonstrated the horizontal and vertical forearm movements that the participant would perform during the experiment and instructed the participant to perform the movements at a self-selected frequency and amplitude, and to maintain the same comfort-mode frequency and amplitude within and across trials. Participants were given approximately 60 s to practice the horizontal and vertical movements and establish their comfort-mode frequency and amplitude.

After participants indicated that they understood the task they completed four control trials: two horizontal and two vertical movement trials. Following the control trials, participants completed 16 experimental trials, eight tracking trials and eight non-tracking trials. For the tracking and non-tracking trials, participant and stimulus motion direction was crossed such that participants completed two horizontal-horizontal, two vertical-vertical, two horizontal-vertical, and two vertical-horizontal trials (Figure 1a). All trials were 45 s in length. After completing the experimental trials a funnel debriefing procedure was performed to determine (1) if participants guessed the true purpose of the study and (2) if they were aware or noticed if their movements became coordinated with the oscillating visual stimulus. None of the participants guessed that the study was investigating movement coordination. Only 3 of the 12 participants noticed that their movements were sometimes coordinated with the visual stimulus, but they all indicated that this coordination was not intentional and that noticing the coordination was not something that they thought influenced or changed how they performed the task. After this, participants were debriefed and thanked for their participation.

### Signal Processing and Measures

The participant and stimulus movement time-series were low-pass filtered using a 10 Hz Butterworth filter and the first 5 s of each trial was removed to eliminate transients.

#### Measures of coordination

Cross-spectral coherence was employed to evaluate the degree to which the participant's instructed and non-instructed movements became spontaneously entrained to the motion of the oscillating stimulus. Additionally, cross-spectral coherence was also used to evaluate the degree of entrainment between the participant's movements in the instructed and non-instructed dimensions. Coherence was calculated between the stimulus motion time-series and both the participant's instructed and non-instructed movement time-series at the peak frequency of the stimulus's movement time-series (i.e., 0.8 Hz). Coherence measures the degree of coordination between two time series on a scale from 0 (no entrainment) to 1 (absolute entrainment). Concerning the relation between movements in the instructed dimension and stimulus movement, we expected more spontaneous entrainment to occur for congruent conditions compared to incongruent conditions and more entrainment for the tracking condition compared to the non-tracking condition. Concerning the relation between movements in the uninstructed dimension and stimulus movements as well as the relation between the participant's movements in both dimensions, we expected spontaneous entrainment to occur for all conditions, but be significantly greater entrainment for the incongruent-tracking condition.

#### Measures of spatial deviation

Principle components analysis (PCA) was used to calculate two measures of spatial deviation on a cycle-by-cycle basis: (1) the mean angular deviation (*Δθ*) from the instructed axis of motion; and (2) the mean normalized variability (*δ*) of the participants' movement about the principle axis of motion. Movement cycles were defined as the period between the maxima (peaks) of the forearm movement in the instructed axis of motion. *Δθ* was determined using PCA by calculating the angle of the first (principle) eigenvector (*v*_1_) from the covariance matrix between x (horizontal) and vertical (y) coordinates for the movement data relative to the instructed axis of motion. *δ* equaled the ratio of the eigenvalue (*λ*) of the second principle axis, *v*_2_, relative to the eigenvalue of *v*_1_ (i.e., *δ = λ*_2_/*λ*_1_). As a normalized ratio of the excursions perpendicular to the principle axis of motion, *δ* provides a measure of movement ‘straightness’ or ‘spread’ relative to the angular direction of motion [see 36 for more details].

**Figure 2:**
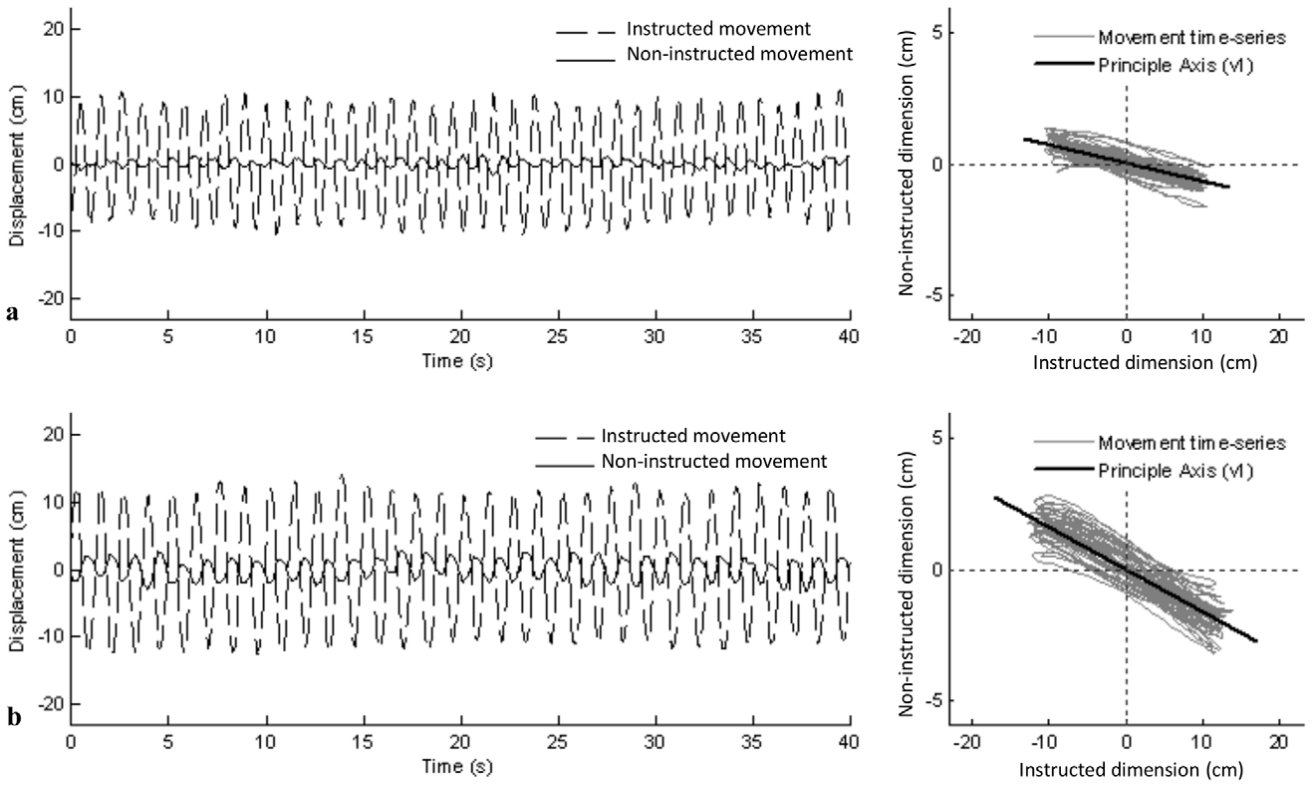
Example movement time series. Both example time-series exemplify the typical instructed and non-instructed movements of a single participant for a (a) congruent and (b) incongruent trial. For both (a) and (b), the instructed movement was performed in the horizontal (i.e., frontal) dimension. The black line on the right panel reflects the principle eigenvector (*v*_1_) calculated using PCA (see text for more details). Even though the two examples shown in the figure have the same angular direction of deviation, both angular directions were observed in the pool of participants. Furthermore, nearly every participant exhibited some degree of angular deviation in all congruent trials.

Prototypical time-series of a participant's instructed and non-instructed movements for congruent and incongruent trials are shown in Figure 2 (both trials are tracking trials in which the participant was instructed to move in the horizontal axis). The adjacent instructed by non-instructed movement plots illustrate the overall predictions for the spatial deviation measures. For *Δθ*, lower values indicate a closer adherence to the instructed movement axis. Thus, we expected that *Δθ* would be greater for incongruent compared to congruent conditions, especially when visual tracking occurs. For *δ*, lower values correspond to a less deviation or variability relative to the actual direction of motion. Accordingly, *δ* should be greater for the incongruent compared to the congruent conditions.

#### Statistical analysis

As no significant differences were found for participant movement direction the congruent horizontal-horizontal and vertical-vertical conditions were combined, as were the incongruent horizontal-vertical and vertical-horizontal conditions. The dependent measures were analyzed using a 2 (congruency: congruent, incongruent)×2 (visual tracking: tracking, non-tracking) repeated-measures ANOVA. Effects sizes are reported as partial eta square (*μ_p_^2^*) and post-hoc analyses were performed using Tukey tests. As the omnibus ANOVA does not allow for a test of whether the experimental measures of movement coordination and spatial deviation were significantly greater than control, planned contrasts were used to compare the control condition to each experimental condition [37].

## Results

### Instructed movement to stimulus relationship

**Figure 3:**
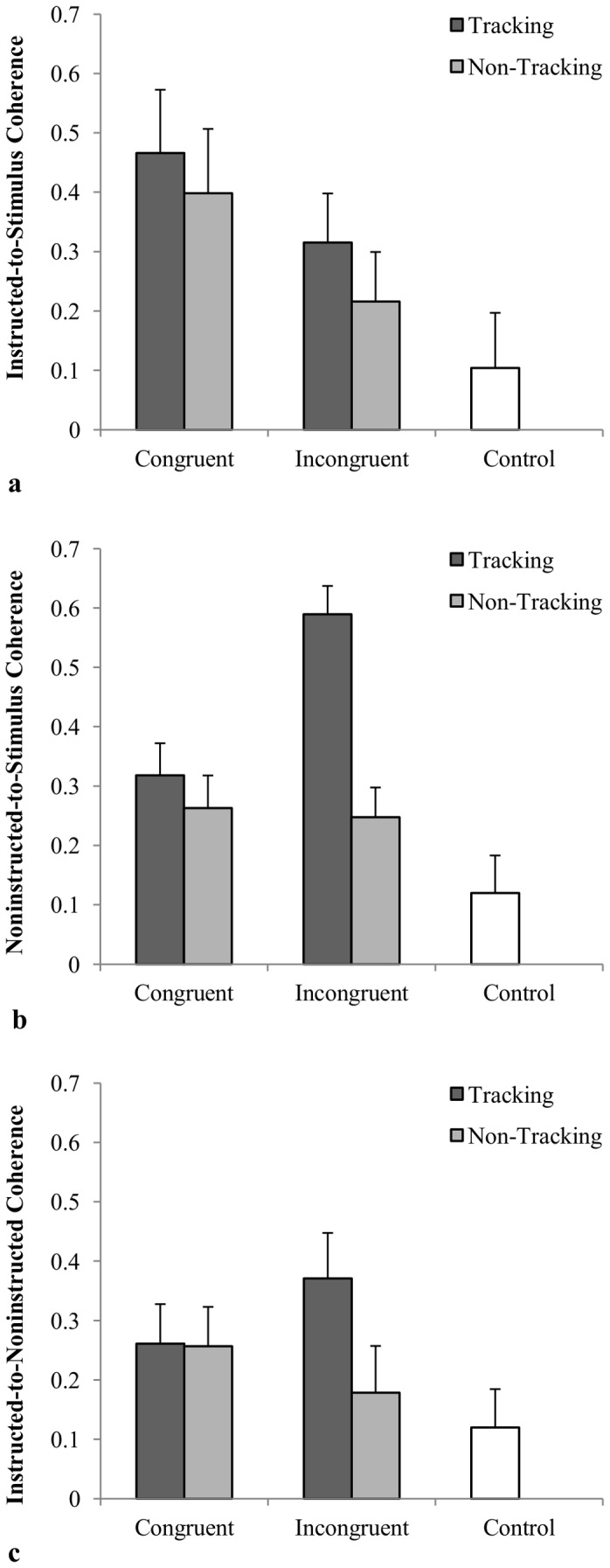
Measures of coordination. Mean cross-spectral coherence between (a) instructed and stimulus, (b) non-instructed and stimulus and (c) participants' instructed and non-instructed movements as a function of tracking and congruency.

As expected, the instructed movements became spontaneously entrained to the movements of the oscillating stimulus (Figure 3a). Accordingly, the planned contrasts revealed that the degree of coherence observed for all of the experimental conditions [all *F*(1, 11)>5.23, *p*<.05, *μ_p_^2^*>.32] was significantly greater than control (chance level coordination), except for the non-tracking-incongruent condition, *F*(1, 11) = 2.28, *p*>.15, *μ_p_^2^* = .17. The ANOVA revealed that there was significantly more coherence observed for the visual tracking compared to the non-tracking condition, *F*(1, 11) = 5.07, *p*<.05, *μ_p_^2^* = .32, and for the congruent compared to the incongruent conditions, *F*(1, 11) = 6.48, *p*<.05, *μ_p_^2^* = .37. There was no tracking by congruency interaction, *F*(1, 11) = <1.

### Non-instructed movement to stimulus relationship

Similar to the instructed-to-stimulus coordination results above, the participants' non-instructed movements became spontaneously entrained to the movements of the oscillating stimulus with significantly greater magnitudes of coherence observed for all of the experimental conditions compared to control [all *F*(1, 11)>5.93, *p*<.05, *μ_p_^2^*>.35]. The ANOVA revealed significant main effects for tracking, *F*(1, 11) = 49.82, *p*<.01, *μ_p_^2^* = .82, and congruency, *F*(1, 11) = 17.44, *p*<.01, *μ_p_^2^* = .61, but more importantly a significant tracking by congruency interaction, *F*(1, 11) = 60.71, *p*<.01, *μ_p_^2^* = .85, with the greatest magnitude of coherence observed for the tracking-incongruent condition (Figure 3b). Indeed post-hoc analysis found that the coherence for the tracking-incongruent condition was significantly greater than the coherence observed for the tracking-congruent, non-tracking-congruent and non-tracking-incongruent conditions (all *p*<.05). There was no difference in coherence between tracking-congruent, non-tracking-congruent and non-tracking-incongruent conditions (all *p*>.05).

### Instructed movement to non-instructed movement relationship

The pattern of coherence results observed for the instructed to non-instructed coordination analysis were very similar to those observed for the non-instructed to stimulus relationship. The magnitude of coherence, however, was markedly less (see Figure 3c) compared to the non-instructed to stimulus relationship. In addition, the magnitude of coherence between the instructed and non-instructed movements was only significantly different form control for the tracking-congruent, tracking-incongruent and non-tracking-congruent conditions [all *F*(1, 11)>5.36, *p*<.05, *μ_p_^2^*>.32], but not for the non-tracking-incongruent condition, *F*(1, 11) = 1.24 *p*>.28, *μ_p_^2^* = .10. The ANOVA revealed a significant main effect for tracking, *F*(1, 11) = 15.23, *p*<.01, *μ_p_^2^* = .58, and a significant tracking by congruency interaction, *F*(1, 11) = 11.08, *p*<.01, *μ_p_^2^* = .52. There was no main effect for congruency, *F*(1, 11)<1. As can be seen from an inspection of Figure 3c, the greatest magnitude of coherence was observed for the tracking-incongruent condition. Post-hoc analysis found that the coherence for the tracking-incongruent condition was, however, only significantly greater than the coherence observed for the non-tracking-incongruent conditions (*p*<.05). There were no other significant differences (all *p*>.05).

### Angular deviation from the instructed plane of motion (Δθ)

**Figure 4:**
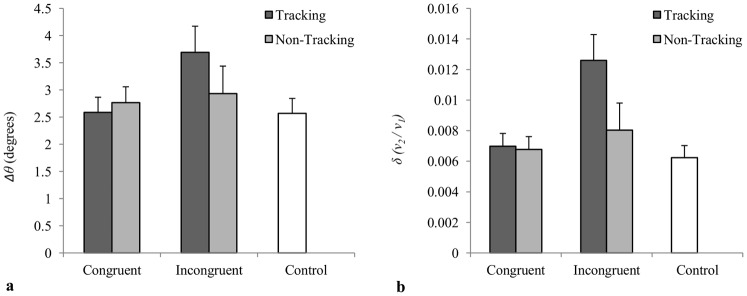
Dependent measures of spatial deviation. (a) Mean angular deviation (*Δθ*) from the instructed axis of motion and (b) mean normalized variability (*δ*) of the participants' movement about the principle axis of motion as a function of tracking and congruency.

As expected, participants deviated away from the instructed axis of motion to a greater degree in the incongruent conditions compared to the congruent conditions, particularly when participants tracked the visual stimulus (Figure 4a). Hence, there was significant main effect for congruency, *F*(1, 11) = 12.49, *p*<.01, *μ_p_^2^* = .53, and a significant tracking by congruency interaction, *F*(1, 11) = 7.66, *p*<.02, *μ_p_^2^* = .41. There was no main effect for tracking, *F*(1, 11) = 2.59, *p*>.13, *μ_p_^2^* = .19. Post-hoc analysis revealed that *θ* was significantly greater for the tracking-incongruent condition compared to the tracking-congruent and non-tracking-congruent conditions (both *p*<.05), but was not significantly greater when compared to the non-tracking-incongruent condition (all *p*>.05). However, the magnitude of *θ* for the tracking-incongruent condition was found to be significantly greater than control, *F*(1, 11) = 4.71, *p* = .05, *μ_p_^2^* = .30, whereas the magnitudes of *θ* for the tracking-congruent, non-tracking-congruent, and non-tracking-incongruent conditions were not significantly different from control [all *F*(1, 11)<1.05, *p*>.3, *μ_p_^2^*<.09].

### Variability about the plane of inclination (δ)

As can be seen from an inspection of Figure 4b, the magnitude of *δ* was greatest for the tracking-incongruent condition. As expected, the analysis revealed a significant interaction between tracking and congruency, *F*(1, 11) = 18.33, *p*<.01, *μ_p_^2^* = .63, with the subsequent post-hoc analysis revealing that *δ* was significantly greater for the tracking-incongruent condition compared to the tracking-congruent, non-tracking-congruent, and non-tracking-incongruent conditions (all *p*<.05). There were also significant main effects for tracking, *F*(1, 11) = 33.07, *p*<.01, *μ_p_^2^* = .75, and congruency, *F*(1, 11) = 19.54, *p*<.01, *μ_p_^2^* = .64. Finally, the magnitude of *δ* for the tracking-incongruent condition was significantly greater than control, *F*(1, 11) = 13.34, *p*<.01, *μ_p_^2^* = .55, whereas the magnitudes of *δ* for the tracking-congruent, non-tracking-congruent, non-tracking-incongruent conditions were not significantly different from control [all *F*(1, 11)<2.34, *p*>.15, *μ_p_^2^*<.18].

## Discussion

The current study was designed to test whether the rhythmic movement interference effect is dependent on the degree to which individuals become spontaneously entrained to the observed movement. As suggested by the Richardson et al. (2009) rhythmic coordination hypothesis, if RMI is the result of recruitment of additional degrees-of-freedom to stabilize a global state of coordination with an incongruent observed movement, then it should only occur when there is entrainment between the participant and the stimulus movements. Support for this rhythmic coordination hypothesis was dependent on three key predictions.

The first prediction was that the instructed movements of participants would become spontaneously entrained to the movements of the oscillating visual stimulus and that the degree of spontaneous coordination would be influenced by the kind of visual tracking. Consistent with this expectation and with previous research on spontaneous visual coordination [e.g., 27], the coherence results revealed that the instructed movements of the participants did become spontaneously entrained to the movements of the oscillating visual stimulus, with the degree of coordination being greater for the tracking compared to the non-tracking condition [29]. Equally important, the degree of coordination was also found to be greater for the congruent compared to the incongruent conditions. This latter finding is consistent with previous research on visual entrainment that has shown how differences in the movement kinematics or oscillatory dynamics of coupled movements decrease the stability and occurrence of visual coordination and entrainment [e.g.], [15], [38,39].

The second prediction was that the non-instructed movements of participants would become spontaneously entrained to the oscillating visual stimulus and that the degree of non-instructed-to-stimulus coordination would be significantly greater for the incongruent-tracking condition. Consistent with this expectation, the coherence results reveal that the non-instructed plane movements of the participants did become spontaneously entrained to the movements of the oscillating visual stimulus. More importantly, the greatest amount of non-instructed-to-stimulus movement entrainment was found in the tracking-incongruent condition. Indeed, the degree of spontaneous non-instructed-to-stimulus movement coordination for the incongruent–tracking conditions was over twice that observed in the other experimental conditions (Figure 4b). These results therefore also suggest that action observation effects may, in part, be attentionally modulated [41], whereby the coordination that is observed between the instructed and non-instructed movements of participants is the result of attending to the stimulus more (i.e. tracking).

**Figure 5:**
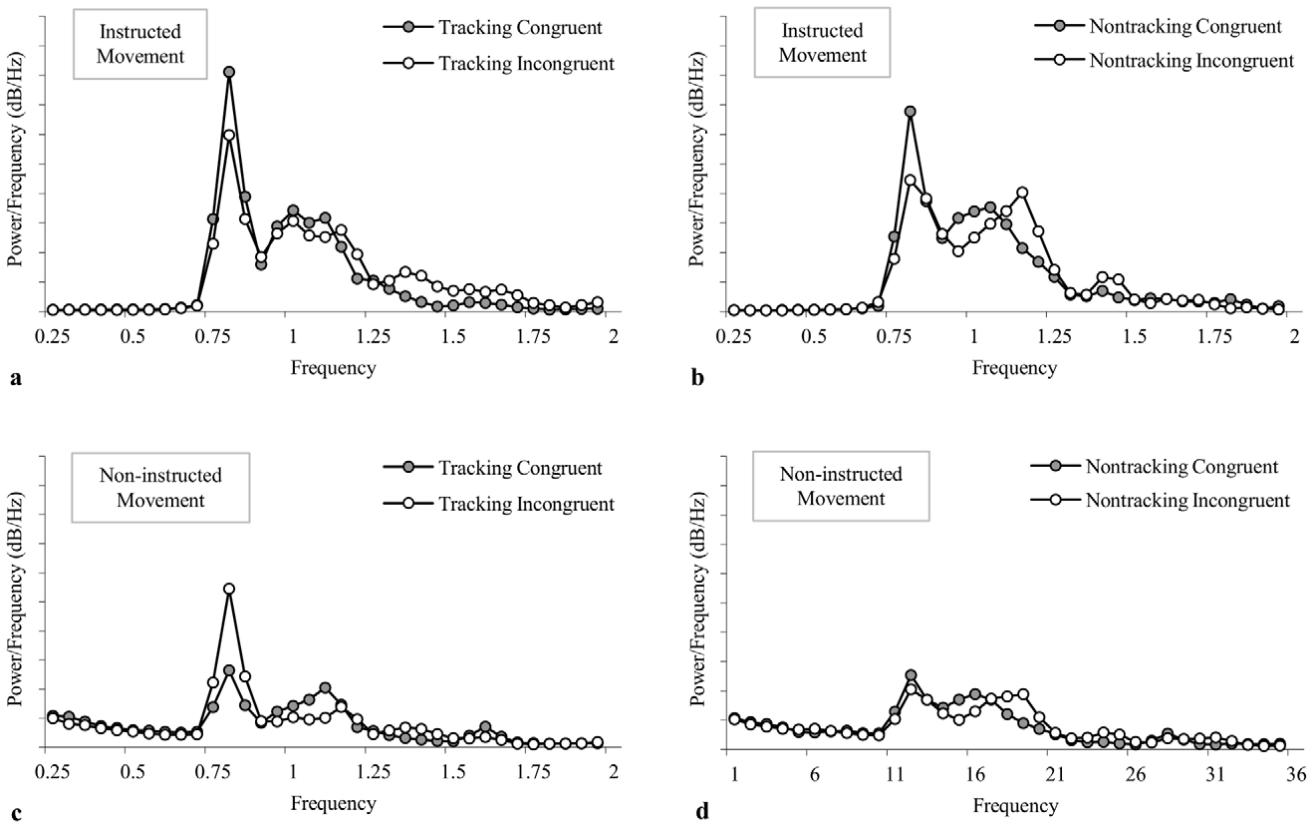
Spectral power distributions. Averaged spectral power distributions of movements in the (a, b) instructed and (c, d) non-instructed axis of motion as a function of tracking and congruency. Spectral power distributions were calculated with frequency bins of 0.05 Hz from 0 to 2 Hz.

To further explore whether the deviation from the instructed axis of movement is a functional and emergent property rhythmic coordination, the entrainment that occurred between the instructed and non-instructed participant movements was also examined. Of particular significance was that pattern of results paralleled those observed for non- instructed-to-stimulus entrainment and, furthermore, that the overall magnitude of the instructed-to-non-instructed entrainment was markedly less than the instructed-to-stimulus and non-instructed-to-stimulus entrainment (see Figure 3). Together, these results indicated that the larger non-instructed angular deviations observed in the tracking incongruent trials was not simply an effect of intrapersonal coordination, but was specific to the task constraints and the non-instructed to stimulus coordination that characterized the tracking-incongruent condition. This latter point can also be discerned from an inspection of Figure 5, in which the spectral power distributions for the instructed and non-instructed movements of the participants for the different experimental conditions are presented. Not surprisingly, these spectral plots reveal that the participants' movements in the instructed axis generally had a peak frequency equal to the frequency of the stimulus (i.e., 0.8 Hz), with the power at that peak frequency consistent with the magnitude of the instructed-to-stimulus coordination observed for the different tracking by congruency conditions (Figure 5a & 5b). More importantly, however, for movements in the non-instructed axis only a notably powerful peak occurred at the frequency of the stimulus during the tracking incongruent trials. This peak in the distribution for the non instructed movement in the tracking incongruent condition is again consistent with the proposal that the non-instructed axis of motion becomes spontaneously employed during instances in which the movements of a participant become entrained with an incongruent stimulus, lending further support for the rhythmic coordination hypothesis.

The final prediction concerned the two spatial deviation measures. Specifically, that the degree of non-instructed movement during an incongruent condition, both in terms of *Δθ* and *δ*, would be a function of the degree to which the instructed and non-instructed movements of participants were coordinated with the visual stimulus. Although dependent on verifying the first two predictions, confirming this last prediction was particularly important. As stated previously, the rhythmic coordination hypothesis predicts that increases in non-instructed movement (i.e., increased spatial deviation) should only occur when a participant becomes coordinated with an incongruent movement. Increased spatial deviation should not occur when simply observing an incongruent movement, as predicted by the standard motor contagion hypothesis. In support of the rhythmic coordination hypothesis, the angular deviation away from the instructed plane of motion (*Δθ*) and magnitude of variability about the angular direction of motion (*δ*) was only found to be significantly greater than control for the incongruent-tracking condition. Recall that of the two incongruent conditions the incongruent-tracking condition was the only condition in which participants exhibited a significantly greater than chance level of instructed-to-stimulus entrainment, as well as the greatest magnitude of non-instructed-to-stimulus entrainment. Thus, not finding a significant increase in spatial deviation for the incongruent-non-tracking condition, in which very little entrainment was observed, also provides support for this last prediction and the rhythmic coordination hypothesis in general.

By finding support for all three of the above predictions, the results of the present experiment demonstrate that the increased non-instructed movement that occurs during the observation of an incongruent movement should not be understood as an “interference effect” or a “movement production error”, but rather an emergent and coherent property of rhythmic movement coordination. These results implicate dynamical processes by which an individual's body and limb movements are mutually responsive to environmental movements in time and space and are emergent properties of a complex system. That is, the dynamic patterns of coordination that exist between produced and observed movements, including the movements involved in the RMI effect, naturally arise from holistic interactions between the neural, biomechanical and perceptual subsystems of the agents and the agents' environment.

It is important to note that the rhythmic coordination and motor contagion hypotheses are not mutually exclusive explanations, and that the motor contagion hypothesis could be adapted to account for the current findings. For example, Blakemore and Frith (2005) made the motor contagion explanation more flexible by stating that when a participant observes an incongruent movement it triggers the motor program associated with it, such that the produced movement is a blend of the intended and the triggered motor programs. We believe, however, that the rhythmic coordination hypothesis provides a more harmonious and functional account for the observed behavior, since it focuses on the behavioral dynamics [42], [19] that underlie complex, goal-directed tasks and not only on the neurocognitive behavior processes. We do not mean to refute the potential importance of neurocognitive processes in perceptual-motor behavior [43], [44], but to simply suggest that neurocognitive processes alone may not provide a full account for the effects of action-observation. As demonstrated here, investigating how the dynamics of a goal-directed action constrain and shape ongoing activity can alter how one understands the perceptual-motor processes that underlie embodied-embedded behavior. That is, understanding of the behavioral dynamics underlying phenomena such as the rhythmic movement interference effect might provide further insights into how the functional organization of the neurocognitive mechanisms implicated in the motor contagion account are fit to the embodied-embedded constraints in which those mechanisms emerge and sustain themselves [45].

Finally, although there have been claims suggesting that the agency or perceived agency of environmental stimuli can affect RMI [10], [12], there is now a growing body of research demonstrating that agency has little to no direct effect on the emergence and qualitative stability of interpersonal and environmental behavioral coordination [10], [46] and that differences in the kinematics or inherent movement properties between observed and produced movements, as well as attention to movement information, may account for previous agency effects [47], [29], [48]. A great deal of the previous research conducted on the rhythmic movement coordination of two spatially congruent movements has also demonstrated how the same coordination dynamics constrain intrapersonal [17], [23], [24] and interpersonal systems [1], [2], [15], [16], [25], [26], [28], [34], as well as systems comprised of one human agent and an environmental stimulus [19], [27], [29], [30], [35]. Consequently, even though the present study employed a computer-generated environmental stimulus it seems reasonable to conclude that the rhythmic coordination hypothesis examined in the current study should provide a generalized account of the perceptual-motor processes underlying rhythmic movement interference during social interactions or any other situation in which an individual is producing a rhythmic movement while observing movement kinematics produced by an intentional agent (also see e.g., [15], [16]).
